# Assessment of the usability of a digital learning technology prototype for monitoring intracranial pressure**^1^**

**DOI:** 10.1590/1518-8345.1054.2777

**Published:** 2016-08-29

**Authors:** Lilian Regina de Carvalho, Yolanda Dora Martinez Évora, Silvia Helena Zem-Mascarenhas

**Affiliations:** 2 MSc, Professor, Serviço Nacional de Aprendizagem Comercial, SENAC, São Carlos, SP, Brazil.; 3 PhD, Full Professor, Escola de Enfermagem de Ribeirão Preto, Universidade de São Paulo, PAHO/WHO Collaborating Centre for Nursing Research Development, Ribeirão Preto, SP, Brazil; 4 PhD, Associate Professor, Departamento de Enfermagem, Universidade Federal de São Carlos, São Carlos, SP, Brazil.

**Keywords:** Educational Technology, Health Technology, Nursing, Evaluation

## Abstract

**Objective:**

to assess the usability of a digital learning technology prototype as a new method for minimally invasive monitoring of intracranial pressure.

**Method:**

descriptive study using a quantitative approach on assessing the usability of a prototype based on Nielsen's ten heuristics. Four experts in the area of Human-Computer interaction participated in the study.

**Results:**

the evaluation delivered eight violated heuristics and 31 usability problems in the 32 screens of the prototype.

**Conclusion:**

the suggestions of the evaluators were critical for developing an intuitive, user-friendly interface and will be included in the final version of the digital learning technology.

## Introduction

Technology has a major influence on current society. Information emerges rapidly from all over the world, and can be accessed via computers and mobile devices connected to the Internet. It is fair to say that technology and society have become inseparable^1^. 

This vast demand for information brings with it professional competition and the demand for increasingly qualified and well educated professionals in all areas of knowledge. In healthcare, continued professional education enables developing competences and skills to make decisions on behalf of patient safety^2^.

However, lack of time due to overload and an excessive number of daily tasks is often an obstacle for continued education. On the other hand, the working environment can facilitate Distance Learning (DL)^2^ ^-^ ^5^, offering flexibility in terms of learning pace and hours^6^ ^-^ ^7^.

DL is expanding in this country, and is considered an efficient tool as it enables anyone to seek knowledge unhindered by national or other borders^5^. Among the tools available, DL is a digital learning technology that has been increasingly used to promote learning in healthcare, especially for nurses^6^ ^,^ ^8^ ^-^ ^10^.

Using digital learning technology could be an efficient strategy in the teaching/learning process. However, developing this tool requires rich and dynamic content, and a well-designed interface that is intuitive to the users^11^. Thus it is important that these resources be evaluated by professionals or users, regardless of the method used.

Numerous methods have been used to evaluate digital learning tools^4^ ^,^ ^11^ ^-^ ^12^, however regarding the quality of the interface, meaning a product that satisfies and meets user demands, the method most often used is usability^13^ ^-^ ^16^. 

Usability assessment normally refers to the ease with which users can perform specific tasks when interacting with the tool or object using the appropriate interface. It is related to five attributes: ease of learning, memorization, mistake prevention, efficiency and user satisfaction^17^.

A systematic review shows that one of the most often used approaches to assess usability is a heuristic evaluation^18^. Among them are Nielsen's heuristics^17^, a simple and low-cost method^19^ that can detect a number of usability problems with a small number of evaluators^15^ ^,^ ^19^ ^-^ ^20^ in a relatively short period of time^21^. This approach provides reliable results and suggestions to improve the interface^16^ ^,^ ^19^ ^-^ ^20^. 

The earlier the assessment, the lower the cost of making changes^17^. Thus, creating prototypes has proven an efficient and economic strategy, as potential problems can be tested for and detected, then corrected before the final product version is launched^11^ ^,^ ^19^ ^-^ ^20^ ^,^ ^22^. 

In this context, to assess the usability of a digital learning technology prototype for a new method for minimally invasive monitoring of intracranial pressure.

## Method

This is a descriptive study using a quantitative approach to assess the usability of a digital learning technology prototype.

Assessment of usability used an analytical approach where expert usability evaluators inspected the interface in search of problems, and suggested ways to improve the interface^17^. While there are numerous approaches for analytical assessment, in this study we chose heuristic evaluation. ^17^

This method is based on using a set of usability principles that guide evaluators as they use an interface in search of problems and shortcomings^17^. 

The guidelines offer a range of heuristics available, however two experts in usability grouped these heuristics into ten, to facilitate evaluations in practice. These are known as Nielsen's Heuristics^17^.

A heuristic evaluation should be done by a small number of evaluators, normally from three to five. Fewer than three is not enough for reliable results, and more than five are not necessary, as the usability problems found become recurring^17^.

Thus, this evaluation was conducted by IT professionals enrolled in the Graduate Program of the Computer Science Department of the Federal University of São Carlos, who met the following inclusion criteria: specialize in Human-Computer interaction and have participated in previous heuristic evaluations. 

Participants were selected using an intentional method, meaning the researcher chose participants based on inclusion criteria, bearing in mind researcher knowledge of the population and its characteristics, providing the conditions for including individuals in the sample^23^. Thus, four potential participants were formally invited by e-mail. Following confirmation that they were willing to participant, a date and place for the first phase of the evaluation was set.

A heuristic evaluation should include three phases: pre-evaluation, evaluation and a session with the evaluators^17^, all of which are described below. 

### Pre-evaluation 

In this phase, the interface is presented to evaluator, along with information about functionality, objectives and standard terminology. 

**Figure 1:**
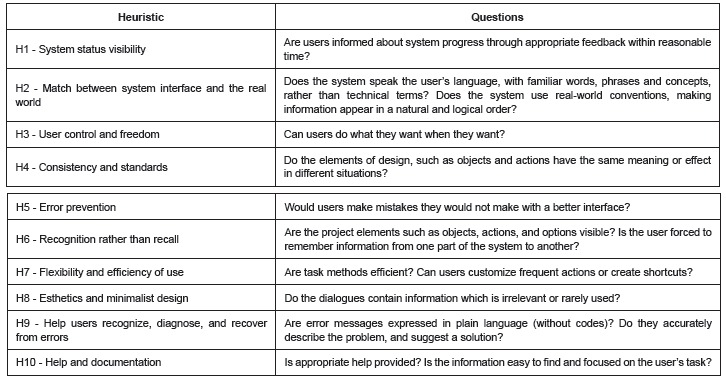
Nielsen's Heuristics^17^ and the questions asked for each one. São Carlos, SP, Brazil, 2013

The prototype was introduced to the evaluators using a Power Point presentation and multimedia projector. At this meeting, which lasted two and a half hours, the researchers and evaluators discussed the standard application of Nielsen heuristics, and the usability problems associated with each one of them, providing everyone with a better understanding of what to evaluate. We distributed a print-out with questions related to each heuristic, as shown in Figure 1. We also asked the evaluators for suggestions of how to address the problems found, which in this type of evaluation is not required. Finally, a date and place for the next meeting was agreed.

### Evaluation

**Figure 2:**
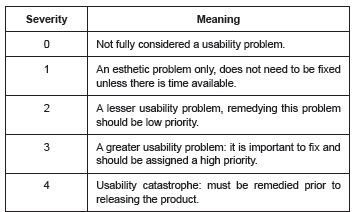
Severity scale based on a usability evaluation using Nielsen's ten Heuristics. São Carlos, SP, Brazil, 2013

This phase is done individually and participants may choose where they want to perform the evaluation. Each evaluator should run through and analyze his/her prototype at least twice. The first run is to make the evaluator familiar with the interface, while the second allows the evaluator to focus on specific elements in search of usability issues using Nielsen's Heuristics^17^, analyzing severity on a scale of zero (0) to four (4), where a higher score is associated with a larger usability problem (Figure 2).

The evaluators chose to use their own computer for individual evaluations. Each one was provided with a CD-ROM containing a prototype and the data collection instrument with the following items: heuristic violated, usability problems, location (prototype screen), severity and suggested solutions. 

### Session with evaluators and project developer.

In this phase the evaluators met with the researcher at the agreed location and date for a meeting lasting three and a half hours. The goal of this meeting was discussion of the evaluations among the evaluators themselves - the heuristics violated, the usability problems found, their severity and location, and suggested solutions for each problem. Evaluators chose do discuss screens in the order in which they appear. Thus each screen was discussed by each evaluator, commenting on the heuristics violated and usability problems found. Where there was disagreement, evaluators discussed until arriving at a formal consensus, which the researcher then entered into the same data gathering tool used for individual assessments. The end result of this phase was a single list of heuristics that had been violated, the usability problems found and the associated severity, the location of the problems and suggestions on how to solve them. 

The project was approved by the UNICEP - Central Paulista University Center Ethics Committee for Research on Human Beings, and abides by domestic and international standards of ethics in research involving human beings. Approval protocol 027/2011. 

## Results

Validation resulted in eight of the ten Nielsen^17^ heuristics violated, and 31 problems in the 32 screens of the prototype (Table 1).

**Table 1:** Violated heuristics, usability problems and their severity in the prototype screens. São Carlos, SP, Brazil, 2013

Heuristic violated	Usability problem		Severity				Total	
	Percent		0	1	2	3	4	
1 - System status visibility	5 (16.13%)				3	2		5
2 - Match between system interface and the real world	7 (22.58%)			3	1	2		6
3 - User control and freedom	7 (22.58%)		1	1	3	2		7
4 - Consistency and standards	5 (16.13%)				2	4		6
5 - Error prevention	3 (9.68%)			1	1	1		3
6 - Recognition rather than recall	1 (3.23%)			1				1
8 - Esthetics and minimalist design	2 (6.46%)			1	1			2
10 - Help and documentation	1 (3.23%)					1		1
Total by heuristic	31 (100%)		1	7	11	12	0	31

The results show that "Correspondence between the system interface and the real world" and "User control and freedom" were the most often violated, with 7 (22.58%) usability problems found in each one, followed by "System status visibility" and "Consistency and standard", totaling 5 (16.13%).

Among the main comments made by the evaluators regarding the violated heuristics, we point out the following: improve the design, with different colors at the header and footer; overly populated screens with too much content; no standard for links; no warning when a user clicks on a link, informing him/her that he/she is on a web page, and proceed if the user does not want to watch the video; no button to turn off sound, which would give users more control over their actions.

The evaluation also found grammar mistakes, limited explanation of the charts and the analogy of a balloon filling up to simulate increased intracranial pressure. 

## Discussion

Technology has been an important tool for healthcare professionals - for their training^3^ ^,^ ^7^ ^,^ ^11^ ^,^ ^24^, the care they give^3^ ^-^ ^4^ ^,^ ^14^ and for research^5^ ^,^ ^18^. In this context, it is necessary to assess these systems, otherwise technology may cease to perform its role as a facilitator, and resources will be under-used. 

They heuristic assessment of this study was performed by four evaluators. While 3 to 5 are recommended for studies of this nature, who may find up to 75% usability problems^15^ ^,^ ^17^ ^,^ ^20^ ^-^ ^22^. We chose to use Nielsen's heuristics^17^, considering them to be the best suited to evaluate software(15,19,22), healthcare information systems^22^ and digital learning tools^16^. 

It is believed that a combination of two or more types of assessment will enable identifying more usability^18^ problems, however Nielsen's heuristics are always present^15^ ^-^ ^16^ ^,^ ^20^. The authors recommend heuristic evaluations and user testing. Heuristic evaluation may be used the first time to identify and correct the more evident problems, and once changes have been made, will also be used for user testing^16^. Thus, as future work in this effort, we intend to evaluate a final version of the prototype with users. 

The results shown show the importance of evaluating a system before it is made available to users. Of the ten heuristics proposed by Nielsen, eight were violated, creating 31 usability problems in the 32 screens of the prototype. Correspondence between system interface and the real world, user freedom and control, system status and consistency and standard accounted for over 77% of the violated heuristics. Studies have shown similar results using the same assessment method as that used in this study^15^ ^-^ ^16^ ^,^ ^21^ ^-^ ^22^.

Usability problems related to these heuristics can reduce the interaction between the system and its users. Therefore, designers must pay attention when designing interfaces, making sure they are intuitive and easy for even an inexperienced user, avoiding additional effort in learning to use the system^25^. 

The project team and the digital learning technology developer quickly understood the suggestions made by the evaluators, as these were solidly based on the heuristics.

Interfaces and content must be simple, and expressed in clear and objective language that is easy to understand^11^ ^,^ ^25^. In one study, the evaluators suggested less content on each page, and that content be arranged by topic. They also suggested using more objective language and a different visual presentation^7^. Excessive content^13^ e and better content organization^12^ were mentioned in other studies. 

Thus, one of the important contributions made by the evaluators was to point out usability problems at the interfaces, which could confuse or distract users, suggesting significant changes to address the problems found. Other studies revealed similar suggestions, such as: insert explanations of charts and images that may not be entirely clear^15^ ^,^ ^25^, a scroll-bar for long texts^25^, changing the color of the interface^9^ ^,^ ^15^ to light green, standardized links^25^, including an error message when users do not correctly fill out the registration screen, stopping wherever the error occurred^16^, and a page describing the icons^25^. In one study evaluating learning technology, evaluators had problems identifying some of the icons^7^, demonstrating how important it is that they be fully intuitive. 

A user-friendly interface may include buttons to make it easier to use. Buttons are used to select items or actions, and should be described using verbs^25^. Here the evaluators suggested a button for users to increase or decrease font size, a button at the footnote to turn off the sound, and a button for printing. Another study led to similar suggestions^16^. 

Another comment made by the evaluators involved user control as they use the interface, which is one of the usability principles. A suggestion was made to use a breadcrumb at the top of the screen to track user actions, showing where the user is and how he/she got there, corroborating another finding^21^ ^)*^.

They "flexibility and efficiency when using" and "help users recognize, diagnose and recover errors" heuristics were not violated. It is believed that these heuristics were not violated as the prototype is not fully functional, and some actions could not be evaluated. Nevertheless, the evaluators made suggestions for developing the final version of the learning technology, such as: "*if the browser is configured not to download images and videos, will content and browsing remain intact, with no loss for the user?*" "*Can the user configure fonts and font size?*"

The least violated heuristics were "recognition rather than recall" *no error message on the registration page* and document support. There is no help item on the pages, and violations related to these heuristics may lead users to an incorrect interpretation of the actual action desired, leading to useless effort, cognitive overload, frustration and dissatisfaction *^_(_^* *^20^* *^-^* *^21^* *^,^* *^25^* *^_)._^*

Severity ranged from 0 (not a usability problem) to 3 (major usability problems), suggesting that some violations are sufficiently serious to warrant attention. None of the violations in this study were considered grade 4 (catastrophic). Each usability problem found in this study, and the associated severity, were taken into consideration, believing they might negatively affect the effectiveness of the final version of the digital learning technology. 

The results of this study show the importance of evaluating digital learning technologies to make them more efficient and user friendly, fostering the teaching and learning process. In nursing, the researchers involved in developing learning systems are concerned with correcting and changing their products based on feedback received from evaluators before making them available to the end user^7^ ^-^ ^10^ ^,^ ^12^ ^-^ ^13^ ^,^ ^24^.

One of the limitations in this study was that the prototype was not fully functional, some aspects could not be evaluated. We also had trouble finding five evaluators fulfilling the inclusion criteria. For that reason we used only four, still within the acceptable for this type of evaluation^17^. 

## Conclusion

Given the findings of this study, we believe the goal was achieved as we were able to identify a large number of usability problems with minimal effort, and using a single type of evaluation.

The advantage of heuristic evaluation is that evaluators who are experts in the method were able to make suggestions for the problems found, highlight the strengths and weaknesses of the project. All of the suggestions were considered in the final version of the digital learning technology.

As future work we intent to evaluate the new version of the digital learning technology with users, as in our view evaluations are essential for providing a quality product.
